# Chikungunya Virus Fidelity Variants Exhibit Differential Attenuation and Population Diversity in Cell Culture and Adult Mice

**DOI:** 10.1128/JVI.01606-18

**Published:** 2019-01-17

**Authors:** Kasen K. Riemersma, Cody Steiner, Anil Singapuri, Lark L. Coffey

**Affiliations:** a Department of Pathology, Microbiology and Immunology, School of Veterinary Medicine, University of California, Davis, California, USA; University of Pittsburgh School of Medicine

**Keywords:** RNA virus, arbovirus, cell culture, chikungunya virus, deep sequencing, fidelity variant, genetic diversity, intrahost virus evolution, mouse, sequencing

## Abstract

Chikungunya virus (CHIKV) is a reemerging global health threat that produces debilitating arthritis in people. Like other RNA viruses with high mutation rates, CHIKV produces populations of genetically diverse genomes within a host. While several known CHIKV mutations influence disease severity in vertebrates and transmission by mosquitoes, the role of intrahost diversity in chikungunya arthritic disease has not been studied. In this study, high- and low-fidelity CHIKV variants, previously characterized by altered *in vitro* population mutation frequencies, were used to evaluate how intrahost diversity influences clinical disease, CHIKV replication, and antibody neutralization in immunocompetent adult mice inoculated in the rear footpads. Both high- and low-fidelity mutations were hypothesized to attenuate CHIKV arthritic disease, replication, and neutralizing antibody levels compared to wild-type (WT) CHIKV. Unexpectedly, high-fidelity mutants elicited more severe arthritic disease than the WT despite comparable CHIKV replication, whereas a low-fidelity mutant produced attenuated disease and replication. Serum antibody developed against both high- and low-fidelity CHIKV exhibited reduced neutralization of WT CHIKV. Using next-generation sequencing (NGS), the high-fidelity mutations were demonstrated to be genetically stable but produced more genetically diverse populations than WT CHIKV in mice. This enhanced diversification was subsequently reproduced after serial *in vitro* passage. The NGS results contrast with previously reported population diversities for fidelity variants, which focused mainly on part of the E1 gene, and highlight the need for direct measurements of mutation rates to clarify CHIKV fidelity phenotypes.

**IMPORTANCE** CHIKV is a reemerging global health threat that elicits debilitating arthritis in humans. There are currently no commercially available CHIKV vaccines. Like other RNA viruses, CHIKV has a high mutation rate and is capable of rapid intrahost diversification during an infection. In other RNA viruses, virus population diversity associates with disease progression; however, potential impacts of intrahost viral diversity on CHIKV arthritic disease have not been studied. Using previously characterized CHIKV fidelity variants, we addressed whether CHIKV population diversity influences the severity of arthritis and host antibody response in an arthritic mouse model. Our findings show that CHIKV populations with greater genetic diversity can cause more severe disease and stimulate antibody responses with reduced neutralization of low-diversity virus populations *in vitro*. The discordant high-fidelity phenotypes in this study highlight the complexity of inferring replication fidelity indirectly from population diversity.

## Abstract (precis)

CHIKV is a reemerging global health threat that elicits debilitating arthritis in humans. There are currently no commercially available CHIKV vaccines. Like other RNA viruses, CHIKV has a high mutation rate and is capable of rapid intrahost diversification during an infection. In other RNA viruses, virus population diversity associates with disease progression; however, potential impacts of intrahost viral diversity on CHIKV arthritic disease have not been studied. Using previously characterized CHIKV fidelity variants, we addressed whether CHIKV population diversity influences the severity of arthritis and host antibody response in an arthritic mouse model. Our findings show that CHIKV populations with greater genetic diversity can cause more severe disease and stimulate antibody responses with reduced neutralization of low-diversity virus populations *in vitro*. The discordant high-fidelity phenotypes in this study highlight the complexity of inferring replication fidelity indirectly from population diversity.

## INTRODUCTION

The global health risk of mosquito-borne chikungunya virus (*Togaviridae, Alphavirus*; CHIKV), which causes a severely debilitating febrile illness marked by polyarthralgia (1), has been highlighted by recent explosive epidemics in the tropics and subtropics (2). While the severity and chronicity of CHIKV-induced polyarthralgia varies, approximately 25% of affected individuals remain symptomatic for two or more months (3). Host and viral factors that drive the severity and duration of disease are not well understood, although autoimmunity (4, 5) and antigen persistence (6, 7) have been implicated. For other RNA viruses, including other arthropod-borne viruses (arboviruses), genetic diversity of the intrahost viral population has been associated with both disease progression (8–11) and tissue tropism (12, 13). However, the role of intrahost CHIKV genetic diversity in chikungunya arthritic disease is unknown.

CHIKV encodes a viral polymerase incapable of proofreading that, coupled with exponential population growth, generates genetically diverse viral populations in hosts (14). RNA viruses like CHIKV are presumed to converge on a replication fidelity that optimizes either the trade-off between adaptability through genetic diversity and the accumulation of deleterious mutations (14, 15) or between replication speed and replication fidelity (16–18). In support of this premise of optimized fidelity, laboratory-generated fidelity-variant viruses replicating with either increased or decreased mutation rates compared to their wild-type (WT) progenitors typically exhibit reduced fitness and virulence (12, 19–32), although counterexamples have been reported. A high-fidelity variant of foot-and-mouth-disease virus was reported to exhibit enhanced fitness *in vitro* (33), and a low-fidelity variant of Venezuelan equine encephalitis virus exhibited virulence comparable to that of WT virus in mice (34). Fidelity variant viruses allow for manipulation of intrahost diversity and can be harnessed to study phenotypic effects of intrahost diversity. For CHIKV, point mutations that arose during *in vitro* mutagen treatment in 2 viral nonstructural genes, *nsP2* and *nsP4*, were shown to confer mutagen resistance and alter mutation frequencies of *in vitro* CHIKV populations in standard arbovirus cell lines (20, 21, 35). High-fidelity CHIKV resulted from substitutions in nsP2 G641D and nsP4 C483Y (here termed double mutant high fidelity, or DM HiFi) (35) or nsP4 C483Y alone (high fidelity, or HiFi) (21), while a low-fidelity phenotype was observed with nsP4 C483G (LoFi) (20).

Previous studies characterized *in vitro* growth kinetics and mutation frequencies for fidelity-variant CHIKV (20, 21, 35). Both DM HiFi (35) and HiFi CHIKV (21) exhibited replication kinetics similar to those of the WT, while LoFi produced higher levels of viral RNA than the WT but similar levels of infectious virions (20). Mutation frequencies, measured by bacterial cloning methods, of viral populations 24 h postinoculation of hamster cells were reduced for DM HiFi (35) and HiFi (21) and elevated for LoFi (20) relative to the WT, leading to their designation as fidelity variants. CHIKV fidelity variant fitness was also assessed in neonatal C57BL/6 mice, in which HiFi CHIKV generated lower infectious virus levels in the blood and liver than WT CHIKV (21) and LoFi CHIKV generated lower CHIKV RNA levels in muscle, blood, brain, and liver than WT CHIKV (20). DM HiFi has not been studied *in vivo*. Despite reversion in mosquitoes, the stability of the LoFi mutation in neonatal mice was not reported (20). Together, these three studies provide evidence that intrahost CHIKV diversity can affect viral fitness in mice. However, since CHIKV-induced polyarthralgia in adult humans is of great public health relevance, evaluation of fidelity-driven modification of disease in an arthritogenic adult mouse model is needed. Additionally, the role of the genome-wide CHIKV mutant spectrum on infection dynamics and disease severity has yet to be defined; previous studies relied on Sanger sequencing of a portion of the CHIKV genome, which produces a lower breadth and depth of sequencing coverage than next-generation sequencing (NGS) approaches (20).

In this study, we describe experimental infection of immunocompetent adult mice with fidelity-variant or WT CHIKV to assess the effects of intrahost CHIKV population diversity on arthritic disease and neutralizing antibody production. We used NGS to compare CHIKV populations between fidelity variants and tissues in infected mice. Inoculation of CHIKV in the footpads of adult mice produces localized arthritis and foot swelling, representing the best murine model of human arthritic disease (36). An immunocompetent adult mouse model also allows for testing neutralizing antibody development, an important protective measure with implications for vaccine development. The high-fidelity mutations used in this study have been proposed as safety enhancers for CHIKV live-attenuated vaccines (LAV) based on the premise that lower mutability reduces the likelihood of reversion to virulence (37).

Based on the rationale that WT CHIKV replication fidelity has evolved to maximize viral fitness, we hypothesized that both high- and low-fidelity CHIKV variants would exhibit reduced fitness, in the form of attenuated replication kinetics and restricted tissue tropism, and would elicit milder arthritic disease. Furthermore, we anticipated that both high- and low-fidelity CHIKV would stimulate lower serum-neutralizing antibody titers than the WT in adult mice. Surprisingly, our results show that the high-fidelity CHIKV variants replicate to titers comparable to those of the WT in adult mice and elicit more severe foot swelling, whereas low-fidelity CHIKV exhibits attenuated replication and foot swelling. NGS revealed that high-fidelity CHIKV populations are more diverse than WT populations in mice, an outcome which we then recapitulated by serial *in vitro* passage. We also found that mouse sera developed against both high- and low-fidelity CHIKV exhibit a diversity-dependent reduction in neutralization of WT CHIKV *in vitro*. Taken together, our findings suggest that the observed diversity of CHIKV populations depends on the cell or host environment they infect and highlight the complexity of inferring fidelity phenotypes from population diversity.

## RESULTS

### CHIKV fidelity variant phenotypes are supported *in vitro* by growth curves and bacterial cloning.

**FIG 1:**
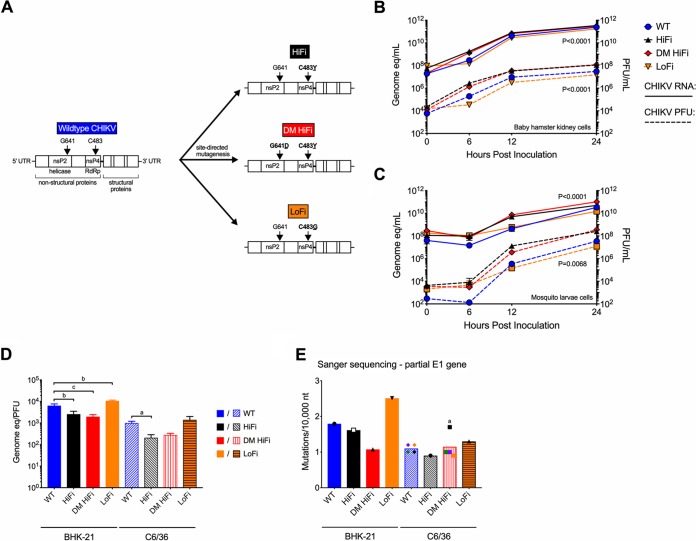
CHIKV fidelity variants replicate to comparable titers and exhibit altered mutation frequencies by bacterial cloning. (A) Schematic showing generation and nomenclature for CHIKV fidelity variants. CHIKV fidelity variants were generated by site-directed mutagenesis of WT CHIKV at nsP2 641 and/or nsP4 483. One-step growth kinetics were determined in baby hamster kidney cells (BHK-21) (B) or *Aedes albopictus* mosquito larva cells (C6/36) (C). CHIKV RNA measurements by qRT-PCR are represented as genome equivalents (eq) per ml, and infectious virus measured by plaque assay is represented in PFU per ml (*n* = 3 biological replicates per group at each time point). (D) Specific infectivity ratios (genome eq to PFU) were calculated at 24 hpi in BHK-21 and C6/36 cells (*n* = 3 per group). (E) Mutations per 10,000 nt were determined by bacterial cloning and Sanger sequencing from CHIKV populations 24 hpi in BHK-21 and C6/36 cells (*n* = 1 per group, except *n* = 4 for WT and DM HiFi in C6/36 cells). For panels B and C, *P* values were calculated by two-way ANOVA. For panel D, *P* values were calculated by ANOVA with Tukey’s *post hoc* test. For panel E, *P* values were calculated with the Grubbs’ outlier test. All error bars represent standard deviations. a, *P* < 0.05; b, *P* < 0.01; c, *P* < 0.001; d, *P* < 0.0001; ns or absence of letter, *P* > 0.05. nsP, nonstructural protein; RdRp, RNA-dependent RNA polymerase.

The CHIKV fidelity variants (Fig. 1A) used for this study were generated and characterized previously (20, 21, 35). We first sought to confirm the established phenotypes in both BHK-21 and C6/36 cells. Both high-fidelity variants replicated to higher titers than the WT (*P* < 0.0001 by repeated-measures analysis of variance [ANOVA]), with the greatest differences observed 6 (*P* < 0.001 by Dunnett’s *post hoc* test) and 12 (*P* < 0.01) h postinfection (hpi) (Fig. 1B and C). In both cell types, the specific infectivities (ratio of genome equivalents to PFU) (Fig. 1D) were lower for both high-fidelity variants and higher for LoFi than for the WT (*P* < 0.01 by Tukey’s *post hoc* test). Mutation frequencies of each fidelity-variant virus and WT were quantified by bacterial cloning and Sanger sequencing, similar to methods originally used to establish these CHIKV mutants as fidelity variants. In BHK-21 cells, HiFi and DM HiFi CHIKV populations had 10% and 40% lower mutation frequencies, respectively, than the WT, and the LoFi CHIKV population had a 40% higher mutation frequency than the WT (Fig. 1E). Similar relationships were observed in C6/36 cells, except for the DM HiFi mutant. Because this was unexpected, we measured the mutation frequencies of DM HiFi and the WT in C6/36 cells in 3 additional biological replicates, comprising approximately an additional 168,000 nucleotides (nt) sequenced. Each of the additional analyses showed that DM HiFi generated a lower mutation frequency than parallel WT replicates. A Grubbs’ outlier test determined the initial elevated DM HiFi mutation frequency value was an outlier (*P* < 0.05), although no methodological differences across replicates explain the outlier. Given that the fidelity genotypes and phenotypes measured here were similar to those previously observed, we proceeded with an infection experiment in adult mice.

### High-fidelity, but not low-fidelity, CHIKV elicits more severe foot swelling than WT CHIKV.

**FIG 2:**
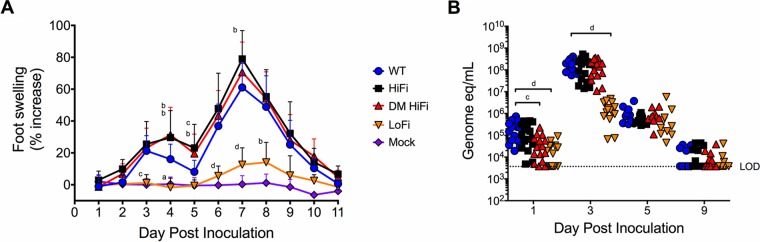
High-fidelity CHIKV produces more severe clinical disease than the WT in adult mice. Adult female C57BL/6J mice were bilaterally inoculated subcutaneously in the rear footpads with 10^3^ PFU of either WT CHIKV, HiFi CHIKV, DM HiFi CHIKV, LoFi CHIKV, or virus-free cell culture supernatant (mock). (A) Bilateral foot swelling was measured as percent increase in dorsoplantar diameter of hind feet from day 0 preinoculation. Numbers of feet per group were the following: for CHIKV cohorts 1 to 3 dpi, *n* = 32; 4 to 9 dpi, *n* = 20; 10 to 11 dpi, *n* = 8; for the mock-inoculated group, 1 to 3 dpi, *n* = 16; 4 to 9 dpi, *n* = 10; 10 to 11 dpi, *n* = 4. (B) Adult mouse viremia titers were determined by qRT-PCR of CHIKV RNA in whole blood. Each symbol represents an individual mouse. LOD is the limit of detection. Error bars represent standard deviations. *P* values for both graphs were calculated by one-way ANOVA. a, *P* < 0.05; b, *P* < 0.01; c, *P* < 0.001; d, *P* < 0.0001; all other cases, *P* > 0.05.

Adult C57BL/6 mice were inoculated with 10^3^ PFU WT or fidelity-variant CHIKV in the rear footpads to test effects of CHIKV fidelity on clinical disease, as determined by rear foot swelling (36, 38–40). Mice infected with LoFi CHIKV exhibited significantly less foot swelling than mice infected with the WT (Fig. 2A) (*P* < 0.05 by one-way ANOVA). Conversely, mice infected with either HiFi or DM HiFi exhibited more severe early footpad swelling than the WT at 3 and 4 days postinfection (dpi) (*P* < 0.01 by one-way ANOVA). CHIKV HiFi-infected mice also exhibited greater peak disease severity than those infected with the WT 7 dpi (*P* = 0.003 by one-way ANOVA). We next evaluated the relationship between clinical disease and viremia (Fig. 2B). Mean viremia titers were significantly reduced in LoFi-infected mice 1 and 3 dpi relative to the WT (*P* < 0.0001 by one-way ANOVA). Lower viremia titers were also observed for DM HiFi-infected mice 1 dpi (*P* < 0.001 by one-way ANOVA) (Fig. 2B), despite elevated clinical disease at later time points. Mean viremias 5 and 9 dpi were not different across groups. These results demonstrate that the high- and low-fidelity mutations elicit more and less severe arthritic disease in adult mice, respectively, and the magnitude of peak CHIKV viremia correlates with disease severity.

### Tissue CHIKV levels are attenuated in LoFi- but not HiFi-infected adult mice.

**FIG 3:**
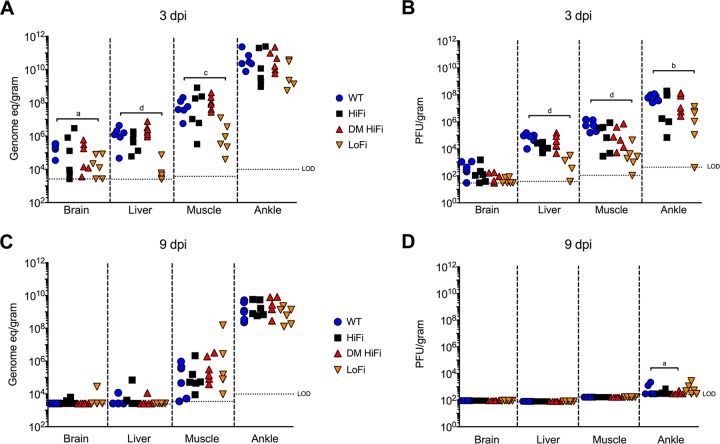
Tissue viral RNA (A and C) and infectious virus (B and D) levels of low-fidelity CHIKV are lower than those of the WT 3 and 9 dpi. CHIKV RNA was assessed by measuring genome equivalents (eq) per gram of tissue, and infectious viruses were measured in PFU per gram of tissue. Each symbol represents an individual mouse, *n* = 6 per group. LOD, limit of detection. LOD varies by tissue based on the mass of tissues tested. *P* values were calculated by two-way ANOVA for all graphs. a, *P* < 0.05; b, *P* < 0.01; c, *P* < 0.001; d, *P* < 0.0001; all other cases, *P* > 0.05.

Infectious CHIKV titers and CHIKV RNA levels in primary target tissues, muscle and ankle, and secondary tissues, brain and liver, were measured to determine whether clinical disease severity was associated with differential viral loads. Similar to viremia kinetics, LoFi CHIKV RNA and infectious virus titers in brain, liver, and muscle were significantly reduced relative to those of the WT 3 dpi (Fig. 3) (*P* < 0.05 by two-way ANOVA). In contrast, HiFi and DM HiFi CHIKV RNA and infectious virus levels were not different from that of the WT in any tissue (*P* > 0.05 by two-way ANOVA). At 9 dpi, low titers (<10^3^ PFU/g) were detected in at least one mouse ankle for all variants, with significantly lower titers in DM HiFi-infected mice than WT-infected mice (*P* = 0.04 by two-way ANOVA). These results indicate that the low-fidelity mutation reduces CHIKV RNA and infectious virus levels in mice but that the high-fidelity mutations do not.

### Intrahost CHIKV mutant spectra vary by individual mouse and tissue.

CHIKV populations from inocula and ankles of 3 mice per treatment group were whole-genome sequenced using Illumina NGS, while sequencing of calf muscle isolates was limited to the whole genome for 1 mouse and partial genome for the remaining 2 mice per group. Sequencing from LoFi-infected muscle was not possible due to poor PCR amplification. For all isolates, the mean depth of coverage postprocessing ranged from 1,126 to 2,622 (Table 1). Comparing mutant spectra of CHIKV isolates from ankles by specific nucleotide substitution frequencies, the only significant differences from the WT were greater frequencies of A>C and G>U substitutions in LoFi CHIKV populations (*P* < 0.01 and *P* < 0.0001, respectively, by two-way ANOVA with Dunnett’s *post hoc* test) (see Fig. 5B).

**TABLE 1:** Descriptive statistics for CHIKV NGS from infected adult mice^*a*^

Virus	Tissue	dpi	Mouse ID	Region of genome	% Covered	Mean depth	Mut freq per 10K	RMSD	Shannon entropy
WT	Inoculum	0	NA	Whole	99.4	2,464	4.01	0.0010	0.0032
HiFi	Inoculum	0	NA	Whole	99.4	2,587	3.92	0.00094	0.0032
DM HiFi	Inoculum	0	NA	Whole	99.4	2,452	4.54	0.0011	0.0036
LoFi	Inoculum	0	NA	Whole	99.4	2,494	3.27	0.00090	0.0027
WT	Tarsus	3	21N	Whole	99.4	2,622	1.74	0.0018	0.0012
WT	Tarsus	3	35N	Whole	99.4	2,380	1.98	0.0012	0.0015
WT	Tarsus	3	35R	Whole	99.4	2,155	2.03	0.00094	0.0016
HiFi	Tarsus	3	25N	Whole	99.3	2,293	2.94	0.0030	0.0019
HiFi	Tarsus	3	25R	Whole	99.4	2,370	2.64	0.0019	0.0019
HiFi	Tarsus	3	31R	Whole	99.4	2,384	2.50	0.0032	0.0017
DM HiFi	Tarsus	3	19L	Whole	99.4	2,479	2.31	0.00085	0.0019
DM HiFi	Tarsus	3	19R	Whole	99.4	2,424	2.42	0.0017	0.0018
DM HiFi	Tarsus	3	33R	Whole	99.3	2,156	2.90	0.0033	0.0020
LoFi	Tarsus	3	23R	Whole	99.2	1,984	4.97	0.017	0.0015
LoFi	Tarsus	3	29N	Whole	99.3	2,468	3.81	0.0077	0.0020
LoFi	Tarsus	3	29R	Whole	99.4	2,388	3.51	0.010	0.0018
WT	Muscle	3	21N	Whole	99.2	1,323	2.20	0.0012	0.0016
WT	Muscle	3	35N	26S-E2	18.3	2,489	2.23	0.0010	0.0017
WT	Muscle	3	35R	26S-E2	18.3	2,555	2.22	0.00085	0.0017
HiFi	Muscle	3	31R	Whole	99.2	1,126	2.42	0.0014	0.0018
HiFi	Muscle	3	25L	26S-E2	18.2	2,213	2.34	0.00083	0.0018
HiFi	Muscle	3	25N	26S-E2	18.3	2,550	2.42	0.0011	0.0018
DM HiFi	Muscle	3	33R	Whole	99.3	2,185	2.53	0.00089	0.0020
DM HiFi	Muscle	3	19L	26S-E2	18.3	2,556	2.11	0.0011	0.0016
DM HiFi	Muscle	3	19R	26S-E2	18.1	1,770	2.60	0.00079	0.0020

a Percent covered, percentage of genome with >300× coverage after read processing; mean depth, mean depth of coverage after read processing; Mut Freq per 10K, mutation frequency per 10,000 nucleotides sequenced. NA, not applicable.

**FIG 4:**
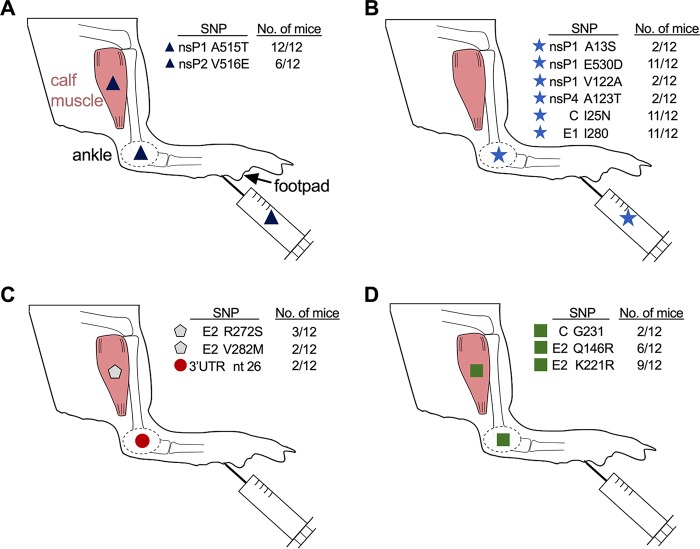
Intrahost mutational spectra vary by mouse and tissue. Single-nucleotide polymorphisms (SNPs) detected in at least 2 mice at frequencies of greater than 1% show 4 patterns of distribution. (A) SNPs detected in inocula, ankle, and muscle. (B) SNPs detected in inocula and ankle but not muscle. (C) SNPs that arose *de novo* (absent from inocula) and detected only in ankle or muscle. (D) SNPs that arose *de novo* and were detected in both ankle and muscle. Nonsynonymous SNPs are listed by residue with amino acid substitution for the gene, while synonymous SNPs list residue with unchanged amino acid. Proportions of mice with each SNP are shown.

Shared SNPs detected in more than one mouse at >1% frequency were identified to characterize tissue- and variant-specific mutations (Table 2). Four trends in the tissue distribution of shared SNPs were observed: (i) SNPs present in inocula and in both ankle and muscle, (ii) SNPs present in inocula and the ankle but not muscle, (iii) SNPs detected in only the ankle or muscle, and (iv) SNPs detected in both the ankle and muscle (Fig. 4). None of the shared SNPs were consensus changes (>50% frequency). The only SNP restricted to a single CHIKV variant was a revertant SNP at nsP4 483, where the LoFi mutant reverted to WT. By 3 dpi in the ankles of LoFi-infected mice, nsP4 483G mutated to 93% G483C in one mouse, 48% G483C and 43% G483V in the second mouse, and 92% G483C and 6% G483V in the third mouse. Valine at nsP4 483 has been previously reported to confer WT fidelity (20). In addition to the consensus changes at nsP4 483, four other consensus changes arose *de novo* in ankles of LoFi-infected mice, but none were detected in any other group of mice. The absence of shared SNPs or consensus changes in HiFi- and DM HiFi-infected tissues suggests that the differences observed in clinical disease and population diversity are unlikely to be the result of particular secondary or compensatory mutations. The four observed distribution patterns show that CHIKV populations are tissue dependent and that SNPs present in inocula can sometimes persist in different tissues.

**TABLE 2:**
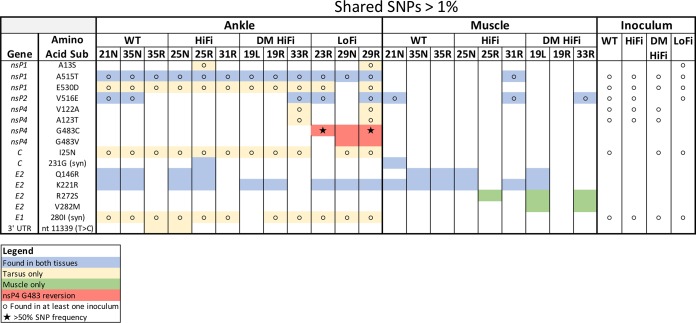
Shared SNPs in mouse tissues and inocula^*a*^

a Highlighted SNPs detected with greater than 1% frequency in at least 2 mice. Mouse identification codes (e.g., 21N), treatment group, and tissue type are reported for each mouse sample. syn, synonymous mutations. The legend details the color coding scheme.

### High- and low-fidelity CHIKV diversify more than the wild type in adult mice.

**FIG 5:**
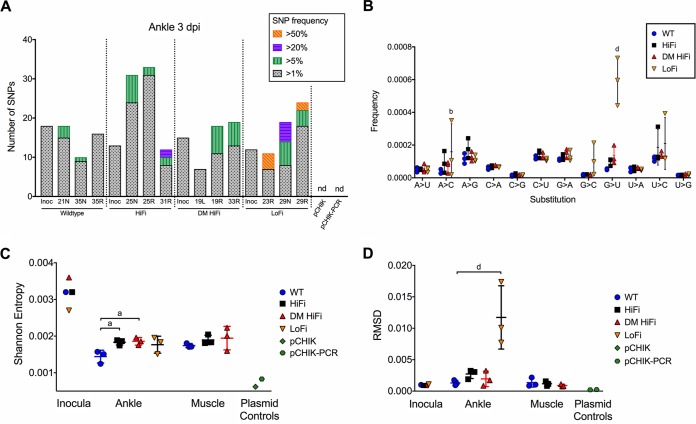
High- and low-fidelity CHIKV diversify more than the wild type in adult mice. CHIKV population diversity was measured by NGS of ankle (whole genome) and muscle (partial genome; LoFi-infected mice excluded) isolates. (A) The number of SNPs across the whole genome in CHIKV populations in ankles was categorized by frequency, where each stacked bar plot represents a CHIKV population from an individual mouse ankle. (B) Mutational spectra for ankle CHIKV isolates at 3 dpi. CHIKV population diversity in ankle and muscle isolates was also measured by Shannon entropy (C) or RMSD (D). Each symbol represents an individual mouse, *n* = 3 per group. Horizontal bars represent mean values, and error bars represent standard deviations. *P* values were calculated by chi-squared test (A), two-way ANOVA (B), and one-way ANOVA (C and D). a, *P* < 0.05; b, *P* < 0.01; d, *P* < 0.0001; all other cases, *P* > 0.05. In panel B, the group with a letter above it was significantly different from the WT. Inoc, inoculum; nd, not detected; pCHIK and pCHIK-PCR, plasmid controls with and without PCR amplicon enrichment.

In addition to comparing mutant spectra, we also compared overall population diversity by number of SNPs (Fig. 5A) and by two proportional diversity metrics, Shannon entropy (Fig. 5C) and root mean square deviations (RMSD) (Fig. 5D). As a metric of virus population genetic variance, RMSD is skewed by high-frequency variants and therefore is useful for comparing high-frequency variants between groups. In contrast, Shannon entropy is maximized at a variant frequency of 0.5 and less biased by variant frequency, so it is better for comparing low-frequency variants between groups. More high-frequency (>5%) SNPs were detected in ankles of HiFi-, DM HiFi-, and LoFi-infected mice, although the differences were not statistically significant (*P* > 0.05 by chi-squared test) (Fig. 5A). Unexpectedly, HiFi and DM HiFi populations in ankles and muscles were comparably or more diverse than WT populations. In ankles, the diversities of HiFi and DM HiFi populations were significantly elevated relative to that of the WT by Shannon entropy (*P* < 0.05 by one-way ANOVA) (Fig. 5C) but not RMSD (*P* > 0.05 by one-way ANOVA) (Fig. 5D). Diversity of LoFi populations was significantly higher than that of the WT by RMSD (*P* < 0.001 by one-way ANOVA) (Fig. 5C) but not Shannon entropy (*P* > 0.05 by one-way ANOVA) (Fig. 5D). In muscle, no differences in population diversities were observed between HiFi and DM HiFi populations relative to the WT by Shannon entropy or RMSD (*P* > 0.05 by one-way ANOVA) (Fig. 5C and D). The pCHIK and pCHIK-PCR sequencing controls had no SNPs at more than 1% frequency (Fig. 5A) and low Shannon entropy and RMSD values (Fig. 5C and D), indicating that false-positive mutations derived from library preparation and NGS had minimal effects on the diversity metrics of CHIKV populations. These results show that relative *in vitro* diversity calculated from mutation frequencies by bacterial cloning and Sanger sequencing does not parallel relative CHIKV population diversities in adult mice, as measured by NGS, or that mutation frequencies detected by these methods change as a function of host environment.

### High-fidelity CHIKV populations diversify more than the WT, as measured by NGS after single and serial passage in vertebrate cells.

**FIG 6:**
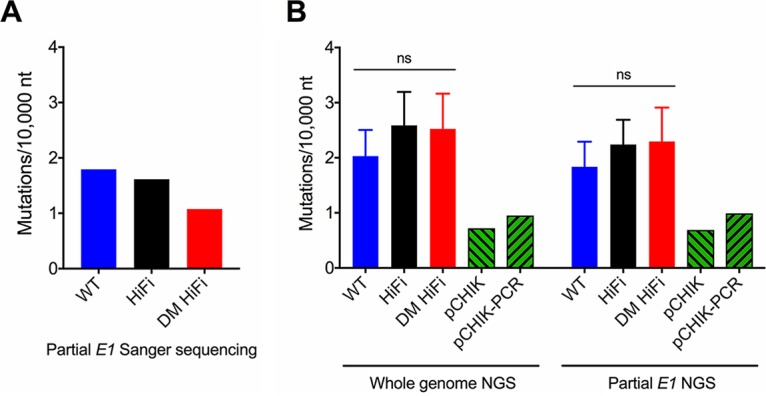
*In vitro* mutation frequencies measured by bacterial cloning and NGS do not align. Mutation frequencies of CHIKV populations were measured after 24 hpi on BHK cells (MOI, 1). (A) Mutation frequencies for WT, HiFi, and DM HiFi populations (*n* = 1) measured by bacterial cloning of 750-nt fragment of *E1*. (Data are the same as those presented in Fig. 1E). (B) Mutation frequencies for WT, HiFi, and DM HiFi populations (*n* = 3) measured by NGS across the whole genome (left) or across the same 750-nt fragment of *E1* as that for bacterial cloning (right). Mutation frequencies of pCHIK and pCHIK-PCR sequencing controls (*n* = 1) are included. Error bars in panel B represent standard deviations. *P* values were calculated by one-way ANOVA for panel B. ns, *P* > 0.05. pCHIK-PCR is CHIKV cDNA with PCR at library preparation (control for PCR error), pCHIK is CHIKV cDNA without PCR as a control for sequencing error.

Despite corroborating HiFi and DM HiFi as high fidelity *in vitro* by bacterial cloning, the unexpectedly expanded diversity of HiFi and DM HiFi populations *in vivo* warranted further investigation. NGS was applied to measure the diversity of CHIKV populations after a single 24-h passage in BHK cells, as was done for bacterial cloning. Mutation frequencies were calculated by NGS across the whole genome and across the same *E1* region used for bacterial cloning. We found that the differences in mutation frequencies established by bacterial cloning were not paralleled by our NGS observations (Fig. 6). Differences in mutation frequencies measured by NGS between both high-fidelity variants and the WT were not statistically significant (*P* = 0.48 by one-way ANOVA) (Fig. 6B). Only small differences were observed between NGS whole-genome and partial *E1* mutation frequencies. The discordance in mutation frequencies measured by bacterial cloning and NGS called into question the attribution of these variants as fidelity variants and prompted further examination of mutation frequencies after serial passage in cell culture.

**FIG 7:**
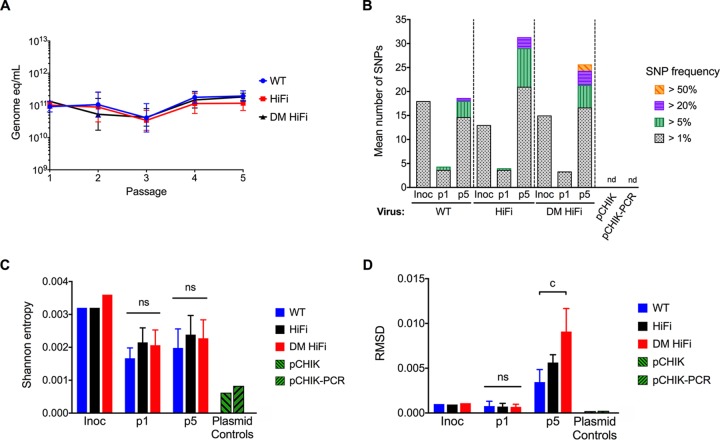
High-fidelity CHIKV variants diversify more than the WT following serial passage. WT, HiFi, and DM HiFi CHIKV were passaged in BHK-21 cells for five serial passages (p1 to p5). p1 and p5 populations were sequenced by whole-genome NGS. (A) CHIKV RNA genome equivalents per ml of cell culture supernatant after each serial passage. (B) Mean number of SNPs across CHIKV genome. (C) Diversity of CHIKV populations as measured by Shannon entropy. (D) Diversity of CHIKV populations as measured by RMSD. Error bars in panels A, C, and D show standard deviations from the geometric mean. *P* values were calculated by repeated-measures ANOVA (A), chi-squared test (B), or one-way ANOVA (C and D). c, *P* < 0.001; absence of letter or ns, *P* > 0.05. Three replicates were passaged, sequenced, and compared per group. One replicate was sequenced for each inoculum.

**FIG 8:**
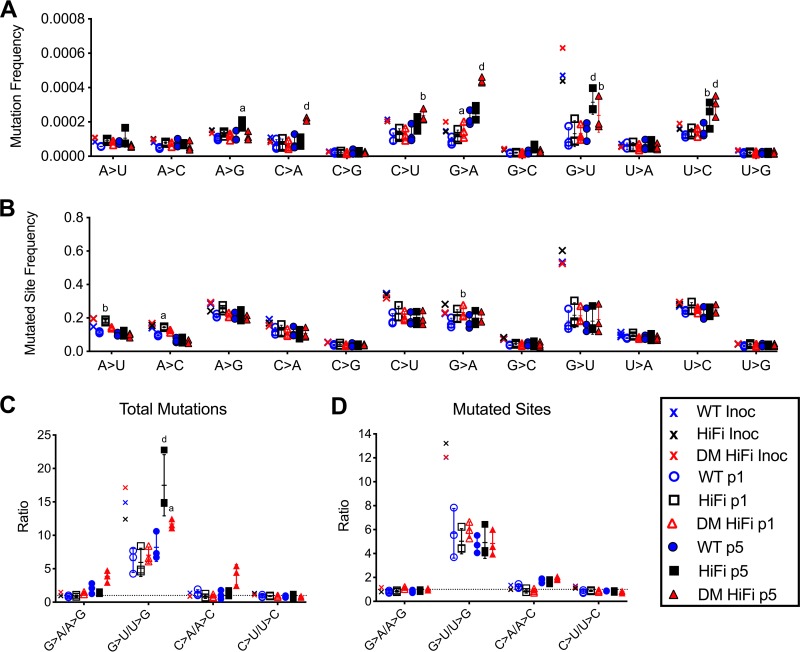
Serially passaged high-fidelity CHIKV populations exhibit mutational biases in total mutations but not mutated sites. Mutant spectra for WT, HiFi, and DM HiFi CHIKV passaged in BHK-21 cells for five serial passages (p1 to p5). Mutational spectra of inocula and passage 1 and 5 CHIKV populations by total mutation frequency (A) and mutated site frequency (B). (C and D) Ratios of GC to AU substitutions by total mutations (C) and mutated sites (D). A mutated site was defined as any nucleotide position with evidence of a substitution regardless of frequency. Error bars in all panels show standard deviations from the geometric means. *P* values were calculated by two-way ANOVA. a, *P* < 0.05; b, *P* < 0.01; c, *P* < 0.001; d, *P* < 0.0001; absence of letter, *P* > 0.05. Three replicates were passaged, sequenced, and compared per group. One replicate was sequenced for each inoculum.

We reasoned that 5 serial passages on BHK-21 cells would amplify real differences in population mutation frequencies. Over serial passages, the viral titers did not vary significantly between passages or virus variants (*P* = 0.95 by two-way ANOVA) (Fig. 7A). The populations from the first passage (p1) and fifth passage (p5) were sequenced by NGS and compared (Fig. 7B to E). The mean depth of coverage for each sample ranged from 2,084 to 2,645 (Table 3). Over five passages, WT CHIKV developed more low-frequency SNPs, while HiFi and DM HiFi developed more high-frequency SNPs (Fig. 7B). RMSDs at p5 were marginally elevated for HiFi and significantly elevated for DM HiFi (*P* = 0.0003 by two-way ANOVA with Tukey’s *post hoc* test) (Fig. 7D). In contrast, Shannon entropy was not significantly different across CHIKV variants at p1 or p5 (Fig. 7C). The elevation in RMSD and accumulation of high-frequency SNPs in both high-fidelity variants relative to WT CHIKV further indicate that HiFi and DM HiFi do not always produce less genetically diverse populations than WT CHIKV. Furthermore, there were no specific nucleotide substitutions in which HiFi and DM HiFi populations had significantly lower frequencies than the WT after p1 or p5 (by two-way ANOVA with Dunnett’s *post hoc* test) (Fig. 8A). After p1, the only significant difference from the WT was a greater G>A frequency for DM HiFi (*P* < 0.05). After p5, HiFi populations exhibited greater frequencies of A>G, G>U, and U>C substitutions (*P* < 0.05, *P* < 0.0001, and *P* < 0.01), and DM HiFi populations had greater frequencies of C>A, C>U, G>A, G>U, and U>C substitutions (*P* < 0.0001, *P* < 0.01, *P* < 0.0001, *P* < 0.01, and *P* < 0.0001). For p5 populations of HiFi and DM HiFi, a trend of GC>AU substitutions at a higher frequency than reciprocal AU>GC substitutions was observed (Fig. 8A and C). For both HiFi and DM HiFi, the G>U/U>G substitution ratios were significantly greater than those for the WT (*P* < 0.0001 and *P* = 0.02, respectively, by two-way ANOVA with Dunnett’s *post hoc* test) (Fig. 8C), while the differences in G>A/A>G and C>A/A>C ratios were not statistically significant. To determine if the GC>AU trend was genome wide, we evaluated the mutant spectra and GC<>AU ratios by frequency of mutated sites (Fig. 8B and D). A mutated site was defined as any nucleotide position with evidence of a substitution regardless of frequency. By mutated site frequency, the observed GC>AU trend is no longer evident (Fig. 8B), and the GC<>AU ratios of p5 populations are similar between HiFi, DM HiFi, and the WT (*P* > 0.05 by two-way ANOVA with Dunnett’s *post hoc* test) (Fig. 8D). These results indicate that the CHIKV fidelity variants in this study did not show differences in mutation bias after 5 serial BHK-21 cell passages.

**TABLE 3:** Descriptive statistics for CHIKV NGS of cell culture-derived samples^*a*^

CHIKV plasmid or variant	CHIKV amplicon enrichment	Overlap error correction	Cell type	Passage no.	Replicate no.	% Covered	Mean depth	Mut freq per 10K	RMSD	Shannon entropy
pCHIKV	No	Yes	NA	NA	1	99.7	2,648	0.72	0.00021	0.00062
pCHIKV	Yes	Yes	NA	NA	1	99.4	2,487	0.95	0.00024	0.00083
pCHIKV	No	No	NA	NA	1	100	28,004	2.06	0.00029	0.002
pCHIKV	Yes	No	NA	NA	1	99.8	58,659	2.03	0.0012	0.0021
WT	Yes	Yes	BHK	Inoculum	1	99.4	2,464	4.01	0.0010	0.0032
HiFi	Yes	Yes	BHK	Inoculum	1	99.4	2,587	3.92	0.00094	0.0032
DM HiFi	Yes	Yes	BHK	Inoculum	1	99.4	2,452	4.54	0.0011	0.0036
WT	Yes	Yes	BHK	1	1	99.4	2,628	2.57	0.0014	0.0020
WT	Yes	Yes	BHK	1	2	99.4	2,452	1.84	0.00046	0.0016
WT	Yes	Yes	BHK	1	3	99.4	2,615	1.68	0.00045	0.0014
WT	Yes	Yes	BHK	5	1	99.4	2,369	3.47	0.0019	0.0026
WT	Yes	Yes	BHK	5	2	99.4	2,528	2.50	0.0037	0.0017
WT	Yes	Yes	BHK	5	3	99.4	2,559	2.55	0.0046	0.0016
HiFi	Yes	Yes	BHK	1	1	99.4	2,515	3.28	0.0011	0.0027
HiFi	Yes	Yes	BHK	1	2	99.4	2,369	2.34	0.00051	0.002
HiFi	Yes	Yes	BHK	1	3	99.4	2,641	2.14	0.00051	0.0018
HiFi	Yes	Yes	BHK	5	1	99.4	2,103	4.88	0.0046	0.0030
HiFi	Yes	Yes	BHK	5	2	99.4	2,626	3.50	0.006	0.0019
HiFi	Yes	Yes	BHK	5	3	99.4	2,468	3.82	0.0062	0.0022
DM HiFi	Yes	Yes	BHK	1	1	99.4	2,274	3.17	0.0010	0.0025
DM HiFi	Yes	Yes	BHK	1	2	99.4	2,084	2.51	0.00057	0.0021
DM HiFi	Yes	Yes	BHK	1	3	99.4	2,632	1.89	0.00046	0.0016
DM HiFi	Yes	Yes	BHK	5	1	99.4	2,259	4.82	0.0061	0.0029
DM HiFi	Yes	Yes	BHK	5	2	99.4	2,508	4.10	0.011	0.0019
DM HiFi	Yes	Yes	BHK	5	3	99.4	2,598	4.38	0.01	0.0020

a Percent covered, percentage of genome with >300× coverage after read processing; mean depth, mean depth of coverage after read processing; Mut Freq per 10K, mutation frequency per 10,000 nucleotides sequenced. NA, not applicable.

### Fidelity mutant CHIKV impair serum neutralization of less diverse WT CHIKV *in vitro*.

**FIG 9:**
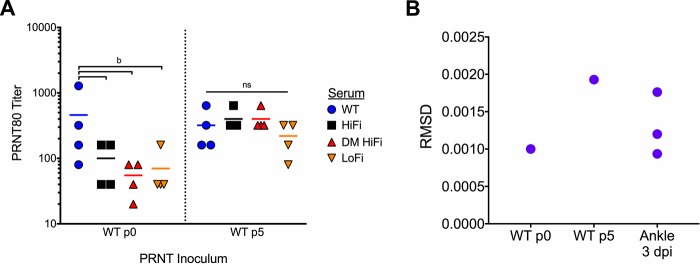
Serum neutralization of wild-type CHIKV slightly impaired by fidelity mutation. Sera from mice 30 dpi with CHIKV WT or fidelity variants were tested for neutralization of WT CHIKV unpassaged (p0) and passage 5 (p5) inocula by PRNT. (A) Each symbol represents the reciprocal of the lowest dilution of mouse serum capable of neutralizing 80% of each PRNT inoculum (PRNT80). (B) RMSD for the p0 and p5 PRNT inocula approximated the minimum and maximum RMSD for WT CHIKV isolated from mouse ankles 3 dpi (the same data are presented in Fig. 5C). For panel A, *n* = 4 mice per group. *P* values were calculated by one-way ANOVA and Dunnett’s multiple-comparison test. b, *P* < 0.01; ns, *P* > 0.05.

Since these high-fidelity mutations are being investigated as safety enhancers for CHIKV LAVs in our other projects, we tested the effect of CHIKV fidelity on neutralization of WT CHIKV populations of low and high relative population diversity. Sera from the four mice in each treatment group at 30 dpi were serially diluted and tested for neutralization of passage zero (p0) and p5 WT CHIKV by PRNT. Sera from WT-inoculated mice neutralized low-diversity p0 WT CHIKV inocula better than any of the fidelity-variant sera (*P* < 0.01 by one-way ANOVA with Dunnett’s multiple-comparison test) (Fig. 9A). In contrast, no differences in neutralization of high-diversity p5 WT CHIKV were observed between any groups (*P* = 0.62 by one-way ANOVA) (Fig. 9A). The RMSD for the low-diversity p0 and high-diversity p5 inocula approximated the minimum and maximum RMSD for WT CHIKV isolated from mouse ankles at 3 dpi (Fig. 9B). The differential neutralization of low- and high-diversity WT CHIKV by sera from fidelity variant-infected mice suggests that serum antibody developed against more diverse CHIKV populations impairs virus neutralization in a diversity-dependent manner.

## DISCUSSION

This is the first study to address effects of CHIKV fidelity mutations on CHIKV-induced arthritic disease. We demonstrate that HiFi and DM HiFi CHIKV replicate faster *in vitro* and elicit more severe arthritic disease in adult mice than the WT while generating viral loads in tissues comparable to those of the WT. Furthermore, we show using NGS that HiFi and DM HiFi CHIKV produce populations with greater diversity than the WT in adult mice, and we reproduce this finding in cell culture. These findings contrast with previous studies reporting comparable *in vitro* replication, reduced mutation frequencies, and attenuated titers of HiFi CHIKV in neonatal mice subcutaneously inoculated in the dorsum (21, 35). The apparent contradiction of high-fidelity CHIKV *in vivo* phenotypes between this study and previous work (21) may be explained in part by methodological differences, as we used adult mice instead of neonates and a 4- to 20-fold higher dose of CHIKV, and we inoculated mice subcutaneously in the rear footpads as opposed to the back. Initiating the infection with more virions and closer to primary target tissues in the foot and ankle joints may allow the virus to overcome transit and tissue barriers to establish infection, overwhelm innate immunity, and resist attenuation. For LoFi CHIKV, our demonstration of attenuated replication in adult mice agrees with the previous report of attenuated replication in neonates (20), suggesting that attenuation of LoFi CHIKV is not host age dependent. Additionally, our observation of genotypic reversion of LoFi to WT in mice, along with the previous report of reversion in mosquitoes (20), further indicates the strong selective pressure against the nsP4 LoFi mutation.

Our initial measures of mutation frequencies using bacterial cloning of *in vitro* populations aligned with the expectation of altered diversity based on the fidelity characterizations, but surprisingly, when we employed NGS, the mutation frequencies for HiFi, DM HiFi, and WT CHIKV were not statistically different after one cell culture passage. To clarify this discrepancy, we serially passaged the CHIKV variants in cell culture to amplify differences in population diversity. After 1 passage, decreased diversity was observed in all CHIKV populations relative to their highly diverse inocula, likely due to purifying selection. After 5 passages, HiFi and DM HiFi populations were more diverse than those of the WT. Furthermore, *in vivo* populations of HiFi and DM HiFi were also found to be more diverse than those of the WT. The increase in diversification of high-fidelity CHIKV populations observed here was counterintuitive but similar to a recent study reporting increased *in vitro* mutation frequencies in populations of Venezuelan equine encephalitis virus, a related alphavirus, bearing an nsP4 mutation analogous to CHIKV C483Y (HiFi) (34).

When measuring intrahost viral diversity, systematic errors (41) and host antiviral deaminases (42, 43) can alter the observed population diversity. To limit systematic errors, we equalized input titers prior to library preparation, prepared libraries in parallel, included CHIKV DNA plasmid libraries as controls for sequencing and reverse transcription-PCR (RT-PCR) errors, and used a conservative quality filter with overlapping read error correction. The absence of called SNPs and relatively low diversity observed in our plasmid controls indicate that the contribution of false-positive mutations from library preparation and sequencer error was minimal. To assess the potential contribution of genetic diversification by antiviral host deaminases, we evaluated mutant spectra for evidence of mutational biases. The mutant spectra of *in vitro* and *in vivo* CHIKV populations in this study lack evidence of specific mutational biases from APOBEC (C>U) (43) or ADAR (A>I) (42) deaminases that could explain the observed differences in population diversity. Instead, the spectra for the *in vitro* p5 populations exhibit an increased frequency of GC>AU substitutions for HiFi and DM HiFi CHIKV. Host adaptation via matched virus-host nucleotide bias has been suggested for RNA viruses (44), including CHIKV (45). To explore this further, we examined the frequency of GC>AU substitution at a nucleotide site level across the genome with the expectation that a shift in GC>AU frequency would result in more G and C nucleotide sites being mutated. Instead, we observed a loss of the GC>AU trend at the nucleotide site level, indicating there was no genome-wide trend but instead that measures of nucleotide bias were skewed by a few sites with high-frequency GC>AU substitutions. In both *in vitro* and *in vivo* CHIKV populations, we observed a higher frequency of transition nucleotide substitutions than transversion substitutions, aside from G>U transversions, as expected (46). The elevated frequency of G>U transversions may be explained in part by oxidative damage during NGS library preparation (47). To assess whether G>U substitutions biased our diversity metrics, we excluded all G>U substitutions, recalculated the metrics, and found the relationships were maintained (data not shown). From these analyses, we conclude that our observed population diversity measurements are unlikely to have been biased by systematic errors, host deaminases, or differential host adaptation. While host adaptation via matched virus-host nucleotide bias is unlikely, we are unable to rule out other intrahost selective pressures that could have affected the observed CHIKV population diversity.

The initial attribution of fidelity phenotypes to the CHIKV nsP2 and nsP4 mutations relied on comparative analysis of mutation frequencies measured by Sanger sequencing and NGS approaches (20, 21, 35). The incongruency in population diversity of high-fidelity CHIKV between this and previous studies highlights the difficulty of inferring replication fidelity from population diversity. To better evaluate potential fidelity-modifying effects of these mutations, future studies using cell-based Luria-Delbruck fluctuation tests (48, 49) and cell-free biochemical assays (50–52) are required to directly measure mutation rates. An additional advantage of these assays is the ability to study effects of the cellular or biochemical environment on fidelity. The type of cell or host has been shown to affect the mutation rate of vesicular stomatitis virus (48) and cucumber mosaic virus (53, 54) and the mutant spectra of human immunodeficiency virus type 1 (HIV-1) (55). Further, the balance or availability of intracellular deoxynucleoside triphosphate (dNTP) pools affects the mutation rate of HIV-1 (56) and spleen necrosis and murine leukemia viruses (57). In this and previous studies (20, 35), cell type has been shown to alter mutation frequencies of CHIKV populations; Stapleford et al. specifically demonstrated that HiFi and DM HiFi replication complexes isolated from cell culture can adjust their replication speed to a greater degree than the WT and utilize low-concentration dNTP pools more efficiently. Whether CHIKV fidelity is determined, as suggested for poliovirus and HIV (18, 58), by the kinetic proofreading model (59–62), which proposes a trade-off between replication speed and accuracy such that accuracy decreases as speed increases, has not been studied. Further studies into kinetic proofreading for CHIKV replication complexes and the effects of dNTP availability on the fidelity phenotype for the nsP2 and nsP4 mutations are warranted. While the incongruency in high-fidelity CHIKV population diversity casts uncertainty on the fidelity phenotypes, we clearly demonstrate that the nsP2 G641D and nsP4 C483Y mutations enhance CHIKV virulence in adult mice.

For a high-fidelity variant of poliovirus, replication speed was suggested to drive attenuation of virulence more so than increased replicase fidelity (18). It is possible that faster replication is driving the enhanced virulence observed here in HiFi- and DM HiFi-infected mice. Stapleford et al. previously showed that isolated HiFi and DM HiFi CHIKV replication complexes synthesize CHIKV subgenomic RNA faster than the WT (35). Here, we demonstrate faster replication of HiFi and DM HiFi CHIKV than of the WT in BHK and C6/36 cells. Furthermore, attenuation of LoFi CHIKV in spite of early reversion to the WT genotype (which we first detected 3 dpi) suggests that robust replication early in infection (<3 dpi) is essential for maximizing peak viral titers and pathogenesis. In the HiFi- or DM HiFi-infected mice, elevated titers in blood early in the course of infection were not observed, but we are unable to compare early CHIKV replication near the inoculation site, as tissues were not collected prior to reaching peak CHIKV titers at 3 dpi. An alternative explanation for the increased virulence not addressed in this study is that the high-fidelity nsP4 C483Y mutation exerts phenotypic effects beyond altered replication speed or fidelity. Unlike CHIKV nsP2 and nsP3, evidence for extensive interactions of nsP4 with host proteins is limited (63), although interactions with proteins of the unfolded protein response within the endoplasmic reticulum have been suggested to promote CHIKV replication (64, 65). Whether nsP4 mutations can modulate the effects of these interactions on viral replication has not been studied. In addition to faster *in vitro* replication, the high-fidelity CHIKV variants counterintuitively produced populations with greater diversity than did the WT. Although the mechanism driving the enhancement in virulence of HiFi and DM HiFi CHIKV remains unclear, the high-fidelity CHIKV variants produce populations with altered diversity relative to those of the WT, a feature that maintains their utility for evaluating intrahost CHIKV evolution.

Our novel characterization of CHIKV population diversity in different tissues by NGS highlights that intrahost CHIKV evolution can be tissue specific. Eight SNPs shared by at least two mice were restricted to ankle or muscle tissues at 3 dpi, with most (6/8) detected in the ankle. Three of the tissue-restricted SNPs arose *de novo*, 2 were nonsynonymous mutations in *E2*, and 1 mutation was detected in the 3′ untranslated region (UTR). Phenotypic characterization of these mutations by reverse genetics is warranted to elucidate their fitness effects. These findings emphasize the value in sequencing from multiple tissues to get a full picture of intrahost populations, as well as the importance of performing NGS on virus inocula to discern *de novo* and preexisting mutations. Our results parallel tissue-specific evolution of poliovirus (66), indicating that tissue microenvironment as a driver of viral evolution is common across RNA virus families.

Use of an immunocompetent adult mouse model also serves as a platform for understanding how the fidelity mutations alter neutralizing antibody responses. Here, we demonstrate a diversity-dependent reduction in serum neutralization *in vitro*, in that CHIKV high-fidelity mutants impair serum neutralization of low-diversity WT CHIKV populations but not high-diversity populations of WT CHIKV. This observation suggests that serum neutralization of CHIKV is driven more by the depth than the breadth of the antibody response, although studies of the antibody repertoire would be required to confirm this idea. Additionally, the presence of specific neutralization-susceptible variants in the p5 WT CHIKV populations biasing serum neutralization titers cannot be ruled out, although the lack of consensus mutations in those populations suggests this is unlikely. Studies are ongoing to address whether the diversity-dependent impairment in neutralization observed here will limit the capacity of anti-high-fidelity CHIKV sera to protect against challenge with WT CHIKV *in vivo*, a better proxy than *in vitro* neutralization. Although the high-fidelity CHIKV populations accumulated greater genetic diversity in serial cell culture, the genetic stability of the nsP2 and nsP4 high-fidelity mutants *in vivo*, in contrast to the unstable low-fidelity nsP4 mutation, suggests they lower the risk of reversion for attenuating mutations, as has been proposed in the context of vaccine development (37).

In summary, we show that the nsP2 and nsP4 high-fidelity mutations induce more severe arthritic disease in adult mice than WT CHIKV while producing more diverse virus populations and serum antibodies less able to neutralize low-diversity inocula *in vitro*. Furthermore, we demonstrate that intrahost CHIKV evolution can be tissue specific. Importantly, our findings highlight the need for direct measurement of replication fidelity to clarify the fidelity phenotype of the nsP2 and nsP4 mutations under different cellular contexts.

## MATERIALS AND METHODS

### Viruses, cells, and viral titration.

Infectious cDNA clones of WT, HiFi, and LoFi 2005 La Réunion CHIKV outbreak strain (06-049; GenBank accession number AM258994.1), generously provided by Marco Vignuzzi, Institut Pasteur, were previously described (20, 21). The nsP2 G641D substitution was introduced by site-directed mutagenesis (QuikChange II site-directed mutagenesis kit; Agilent) in the HiFi CHIKV clone with a single point mutation (GGC>GAC) to generate DM HiFi CHIKV. Genotypic integrity was verified by whole-genome Sanger sequencing for all clones. Infectious CHIKV was rescued from cDNA clones as previously described (15). For rescued virus stocks and experiments described below, viral RNA and infectious virions were titrated in triplicate by quantitative RT-PCR (qRT-PCR) (CHIKV primers 6856, 6981, and 6919-FAM) and Vero plaque assays, respectively, as previously described (67, 68). Baby hamster kidney cells (BHK-21; ATCC CCL-10) and African green monkey kidney cells (Vero; ATCC CCL-81) were maintained in high-glucose Dulbecco’s modified Eagle medium (DMEM; Gibco, Thermo Fisher Scientific) supplemented with 10% fetal bovine serum (FBS; Gibco, Thermo Fisher Scientific) and 1% penicillin-streptomycin (P/S; Gibco, Thermo Fisher Scientific) at 37°C and 5% CO_2_. The *Aedes albopictus* cell line C6/36 (ATCC CRL-1660) was maintained in Schneider’s insect medium (Caisson Labs) supplemented with 20% FBS and 1% P/S at 28°C and atmospheric CO_2_.

### *In vitro* growth assays and serial passage.

BHK-21 and C6/36 cells were inoculated with rescued stocks (p0) of each CHIKV fidelity variant or WT in triplicate at a multiplicity of infection (MOI) of 1. Cell culture supernatants were harvested (1/20 total volume) and replenished after a 1-h absorption period and 6, 12, and 24 h postinoculation (hpi). To amplify differences in mutation frequencies, the WT and the high-fidelity CHIKV variants were inoculated and passaged 5 times in BHK-21 cells in triplicate at an MOI of 1. After 24 hpi, cell culture supernatant was collected and CHIKV RNA titers were used to estimate the PFU titer based on measured genome/PFU ratios at 24 hpi for each variant in the *in vitro* growth assay. Passaged CHIKV supernatants were adjusted to an MOI of 1 for subsequent inoculations.

### Mutation frequencies by bacterial cloning.

Mutation frequencies were measured from CHIKV populations harvested after the first 24-h passage described above. Viral RNA was extracted (Qiagen QiaAMP viral RNA minikit), and a 750-nt region (nt 10019 to 10768) of the *E1* envelope gene was amplified by high-fidelity RT-PCR (Agilent Accuscript PfuUltra II RT-PCR kit). Bacterial cloning of *E1* amplicon cDNA, Sanger sequencing, and mutation frequency calculations were performed as previously described (21). At least 80 amplicons, representing >60,000 nt, were sequenced for each virus from both BHK-21 and C6/36 cells. Since the goal of this step was to validate established genotypes, mutation frequencies were measured by bacterial cloning for one biological replicate per virus in BHK-21 and C6/36 cells, except for WT and DM HiFi in C6/36 cells, where four biological replicates were used.

### Mouse infections.

All research animals were housed at animal biosafety level 3, and procedures were performed in accordance with University of California (UC) Davis IACUC protocol 19108. Six-week-old female C57BL/6J mice (The Jackson Laboratory) were used in this study. Only female mice were used, since the immunocompetent, adult mouse footpad model was described in female C57BL/6 mice only (36), and no sex bias in clinical disease or pathology was reported following footpad inoculation of immunodeficient C57BL/6 adult mice (69). Sixteen mice in each group were bilaterally inoculated subcutaneously in the rear footpads with 10^3^ PFU CHIKV per footpad in 10 μl of sterile 0.9% NaCl solution. Mock-treated mice were inoculated with virus-free cell culture supernatant diluted in 0.9% NaCl solution. Blinded hind foot height measurements were recorded daily by digital caliper operated by the same person. Blood was collected on 1, 3, 5, 9, and 30 dpi, with 30-dpi blood processed to harvest serum. Mice from each treatment group were euthanized on 3, 9, or 30 dpi, and the brain, liver, calf muscles, and ankle joints were collected. Tissues for virus titration by plaque assay were homogenized and stored at −80°C. Tissues for NGS and qRT-PCR were immersed in RNAlater (Thermo Fisher Scientific) at 4°C for 1 day prior to homogenization. RNA was extracted by a MagMax-96 viral isolation kit (AM1836; Thermo Fisher Scientific) on a MagMax Express-96 particle processor (Thermo Fisher Scientific).

### PRNT.

Neutralizing antibody levels from 30 dpi mouse sera were determined by plaque reduction neutralization tests (PRNT) using low-diversity p0 and high-diversity p5 BHK-21 cells with WT CHIKV. Mouse sera were heat inactivated at 56°C for 30 min. Neutralization assays with 2-fold dilutions of mouse sera (1:20 to 1:2,560) were performed using Vero cells as previously described (70). The reciprocals of the highest dilution of sera that caused >80% reduction in plaque formation are reported.

### Amplicon library preparation and next-generation sequencing.

For *in vitro* serial passage isolates, all replicates were sequenced. For CHIKV from mouse tissues, 3 mice with tissue RNA levels at or near the median for each treatment group were selected for NGS. Target enrichment on equivalent quantities of viral RNA was performed by high-fidelity RT-PCR (Accuscript PfuUltra II) of nine cDNA amplicons spanning the CHIKV 5′ to 3′ UTRs (Table 4). For calf muscle, sufficient whole-genome RT-PCR amplification was achieved for just one mouse in the WT, HiFi, and DM HiFi cohorts. For 2 mice in those cohorts, amplification was only achieved with primer set 7, which covered the 26S promoter to the 3′ end of the envelope protein 2 gene (*E2*). Due to poor RT-PCR amplification, NGS was not performed for any LoFi-infected muscle isolates. Amplicons were fragmented with double-stranded DNA fragmentase (New England Biolabs), followed by KAPA pure bead (Kapa Biosystems) size selection targeting a mean length of 150 bp. Library preparations were performed with a NEBNext Ultra DNA library preparation kit and NEBNext multiplex oligonucleotides (New England Biolabs). Libraries generated from CHIKV infectious clone DNA with (pCHIK-PCR) or without (pCHIK) PCR amplification prior to library preparation were incorporated as controls for PCR and sequencing errors. Libraries were sequenced on a single flow cell lane using paired-end 150 Illumina HiSeq 4000 technologies at the UC Davis DNA Technologies Core.

**TABLE 4:** Primers used to generate amplicon libraries for next-generation sequencing

Primer set	Forward primer	Forward primer sequence	Reverse primer	Reverse primer sequence	Annealing temp (°C)
1	15F	CACACGTAGCCTACCAGTTTC	1601R	CCCGCTCTGTCCTCAAGCTG	60
2	1417F	GAGGACTAGAATCAAATGG	2807R	GATGCGGCTGCTGTCATGAC	55
3	2480F	GTGCGGCTTCTTCAATATG	4151R	CACTGTTCTTAAAGGACTC	55
4	3982F	ACTGAATGCAGCCTTCGTAG	5423R	GAACGTCTCGCTTGATGCT	60
5	5041F	AGCGTTGATGGCGAGATAC	6568R	CCACATAGGTATGCTGTCGCC	60
6	6444F	CACTACAGGAAGTACCAATGG	7706R	GTTTGGGTTGGGATGAACT	55
7	7570F	GCAGAAGCCGACAGCAAGTA	9736R	GCCATACCCACCATCGACAG	60
8	9486F	CAACGAGCCGTATAAGTATTGG	10726R	CGCTCTTACCGGGTTTGTTG	60
9	10418F	TTCAGCCTGGACACCTTTC	11756R	GGAAGAGTTCGGTATGCTATG	60

### Bioinformatics.

Demultiplexed paired-end reads were quality (>Q35), adapter, and primer trimmed with Trimmomatic (v0.36) (71). Overlapping paired-end reads were merged, and mismatched base calls were resolved by highest quality score with FLASH (v1.2.11) (72). Reference-guided alignment was performed with the Burrows Wheeler alignment tool (bwa mem, v0.7.5) (73). To control for variance in within-genome and between-sample coverage depth, the aligned reads were downsampled with BBTools (v34.48; Joint Genome Institute) to ca. 2,500× coverage. SNPs were called by LoFreq* (v2.1.2) (74) and annotated with SNPdat (v1.0.5) (75). Shannon entropy was calculated in R (v3.4.3) via the diverse package (76), while RMSD (77) and specific nucleotide substitution frequencies were calculated via in-house R scripts. A minimum coverage cut-off of 300 was used for all analyses to eliminate bias of low-coverage positions. These NGS and bioinformatics methods capture nucleotide substitutions but are biased against deletions and recombination events.

### Statistical analysis.

All statistical analyses were performed with GraphPad Prism 7 software (GraphPad Software, CA, USA). Statistical significance was ascribed to *P* values of less than 0.05.

### Accession number(s).

Raw NGS data are available from the NCBI Sequence Read Archive under BioProject entry PRJNA453810. Pipeline and in-house scripts are available at https://github.com/kasenriemersma/CHIKV-NGS-diversity.
